# Supplementary material for: A network dynamical simulation model for the study of antibiotic resistance in microbial communities
Source: Gut Microbes. 2026 Sep 19;18(1):2734703. doi: 10.1080/19490976.2026.2734703 (PMC13596919; doi:10.1080/19490976.2026.2734703)
Supplement: Supplementary file 1 [file KGMI_A_2734703_SM9053.zip › supplementary_file_1/Figures/ws_delta_gc_frac_n100.pdf]

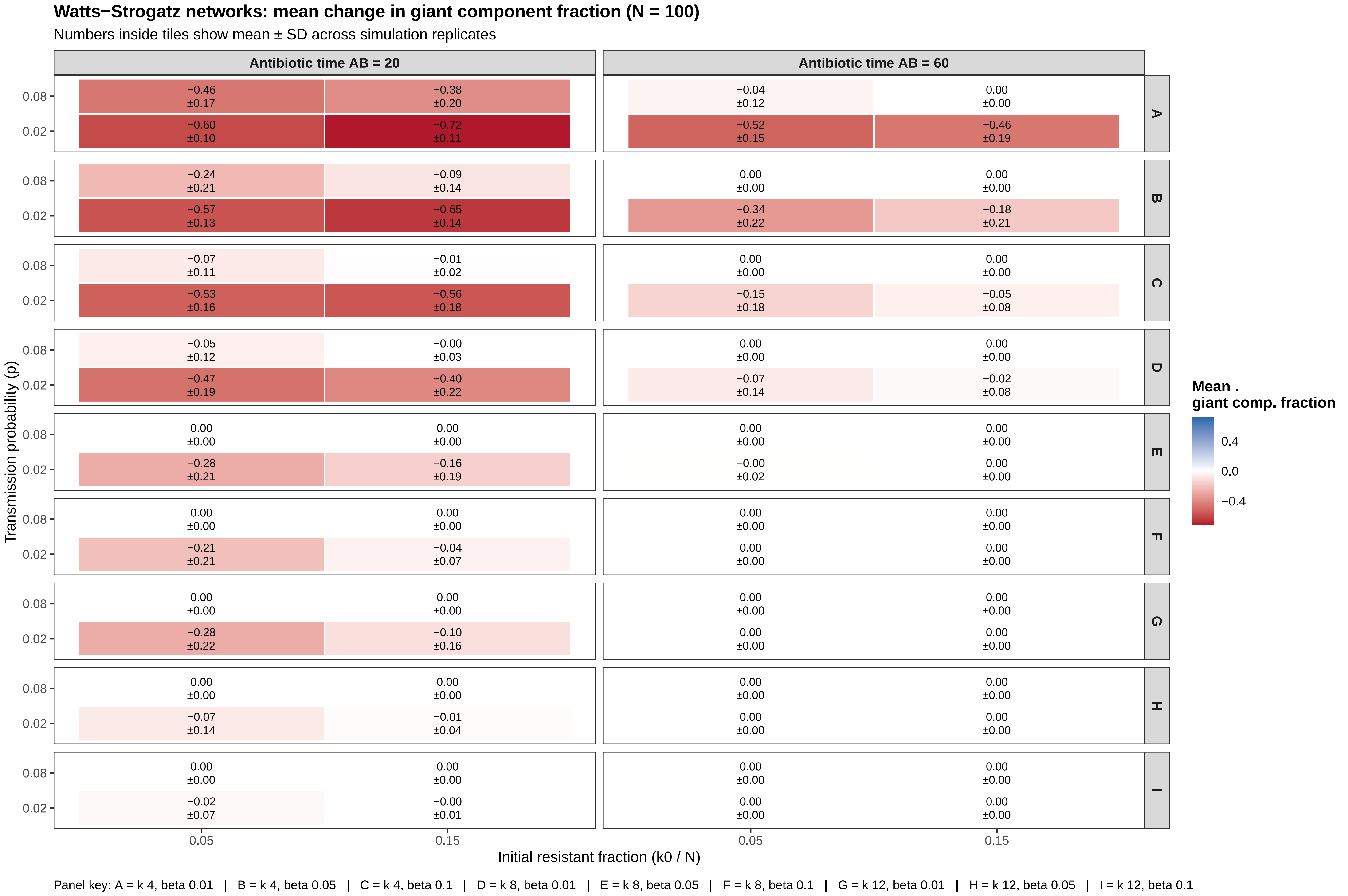
Watts−Strogatz networks: mean change in giant component fraction (N = 100)
Numbers inside tiles show mean ± SD across simulation replicates
Antibiotic time AB = 20
Antibiotic time AB = 60
A
−0.46 ±0.17
−0.38 ±0.20
−0.60 ±0.10
−0.72 ±0.11
−0.04 ±0.12
0.00 ±0.00
−0.52 ±0.15
−0.46 ±0.19
B
−0.24 ±0.21
−0.09 ±0.14
−0.57 ±0.13
−0.65 ±0.14
0.00 ±0.00
0.00 ±0.00
−0.34 ±0.22
−0.18 ±0.21
C
−0.07 ±0.11
−0.01 ±0.02
−0.53 ±0.16
−0.56 ±0.18
0.00 ±0.00
0.00 ±0.00
−0.15 ±0.18
−0.05 ±0.08
D
−0.05 ±0.12
−0.00 ±0.03
−0.47 ±0.19
−0.40 ±0.22
0.00 ±0.00
0.00 ±0.00
−0.07 ±0.14
−0.02 ±0.08
E
0.00 ±0.00
0.00 ±0.00
−0.28 ±0.21
−0.16 ±0.19
0.00 ±0.00
0.00 ±0.00
−0.00 ±0.02
0.00 ±0.00
F
0.00 ±0.00
0.00 ±0.00
−0.21 ±0.21
−0.04 ±0.07
0.00 ±0.00
0.00 ±0.00
0.00 ±0.00
0.00 ±0.00
G
0.00 ±0.00
0.00 ±0.00
−0.28 ±0.22
−0.10 ±0.16
0.00 ±0.00
0.00 ±0.00
0.00 ±0.00
0.00 ±0.00
H
0.00 ±0.00
0.00 ±0.00
−0.07 ±0.14
−0.01 ±0.04
0.00 ±0.00
0.00 ±0.00
0.00 ±0.00
0.00 ±0.00
I
0.00 ±0.00
0.00 ±0.00
−0.02 ±0.07
−0.00 ±0.01
0.00 ±0.00
0.00 ±0.00
0.00 ±0.00
0.00 ±0.00
0.08
0.02
Transmission probability (p)
0.05
0.15
Initial resistant fraction (k0 / N)
Mean .
giant comp. fraction
0.4
0.0
−0.4
Panel key: A = k 4, beta 0.01 | B = k 4, beta 0.05 | C = k 4, beta 0.1 | D = k 8, beta 0.01 | E = k 8, beta 0.05 | F = k 8, beta 0.1 | G = k 12, beta 0.01 | H = k 12, beta 0.05 | I = k 12, beta 0.1
